# Use of fecal volatile organic compound analysis to discriminate between non-vaccinated and BCG—Vaccinated cattle prior to and after *Mycobacterium bovis* challenge

**DOI:** 10.1371/journal.pone.0179914

**Published:** 2017-07-07

**Authors:** Christine K. Ellis, Somchai Rice, Devin Maurer, Randal Stahl, W. Ray Waters, Mitchell V. Palmer, Pauline Nol, Jack C. Rhyan, Kurt C. VerCauteren, Jacek A. Koziel

**Affiliations:** 1 United States Department of Agriculture, Animal Plant and Health Inspection Service, Wildlife Services, National Wildlife Research Center, Fort Collins, Colorado, United States of America; 2 Department of Agricultural and Biosystems Engineering, Iowa State University, Ames, Iowa, United States of America; 3 United States Department of Agriculture, Agricultural Research Service, National Animal Disease Center, Ames, Iowa, United States of America; 4 United States Department of Agriculture, Animal Plant and Health Inspection Service, Veterinary Services, Wildlife Livestock Disease Investigations Team, Fort Collins, Colorado, United States of America; Public Health England, UNITED KINGDOM

## Abstract

Bovine tuberculosis is a zoonotic disease of global public health concern. Development of diagnostic tools to improve test accuracy and efficiency in domestic livestock and enable surveillance of wildlife reservoirs would improve disease management and eradication efforts. Use of volatile organic compound analysis in breath and fecal samples is being developed and optimized as a means to detect disease in humans and animals. In this study we demonstrate that VOCs present in fecal samples can be used to discriminate between non-vaccinated and BCG-vaccinated cattle prior to and after *Mycobacterium bovis* challenge.

## Introduction

Bovine tuberculosis (bTB), a zoonotic disease affecting international public health, agricultural, wildlife, and trade [1, 2], is caused by *Mycobacterium bovis*. The etiological agent responsible for most of the 9.6 million annual human tuberculosis cases [3] is *M*. *tuberculosis*; however, approximately 30% of cases may be caused by *M*. *bovis* infection, especially in developing countries [4] where prevalences of livestock bTB may approach 10–14% [5–8]. The economic costs associated with bTB are significant; from 2008–2009 estimated economic losses globally and in the United States (US) were $3 billion and $40 million, respectively [2].

The US federal bTB eradication program has been successful in decreasing the prevalence of bTB to approximately 0.0006% [2]; however, outbreaks in domestic cattle (*Bos tarus*) and captive and farmed cervids continue to occur. Importation or migration of infected animals from other countries and endemically infected feral swine (*Sus scrofa*; Molokai Island, Hawaii) and white-tailed deer (*Odocoileus virginianus*; Michigan and Indiana) serve as sources for reintroduction of the disease to domestic livestock [9–15]. Antemortem surveillance tests used for domestic cattle include the caudal fold (CFT) and comparative cervical (CCT) skin tests, and the interferon gamma (IFN-γ) release assay (IGRA; Bovigam, Prionics Ag, Schlieren-Zurich, Switzerland). The sensitivity (SN) and specificity (SP) of these tests are reasonable, but all are labor intensive and lack efficient time to results [1, 7]. Other *in vitro* assays have limitations associated with accuracy and execution that restrict their use in ante-mortem surveillance [7].

Development of an ante-mortem disease monitoring system capable of detecting *M*. *bovis* infection in cattle, cervids such as white-tailed deer, feral swine, and other wildlife species would improve bTB surveillance by decreasing or eliminating animal handling events, and remove the requirement for specialized training. This system should be highly repeatable, cost efficient, highly sensitive and specific, and provide rapid and early detection of diseased individuals. Evaluation of volatile organic compounds (VOCs) present in breath, biofluids such as urine and blood, and feces have been conducted in an effort to develop a diagnostic modality for identifying disease (including tuberculosis) in humans [16–27]. In cattle, VOC analysis of breath samples has been explored as a method for diagnosis of bovine respiratory disease [28], brucellosis [29], bTB [30–32], Johne’s disease [29, 33], and ketoacidosis [34, 35]. Fecal VOC analysis has been used experimentally to identify goats [33, 36] and cattle [37] infected with *Mycobacterium avium paratuberculosis* (MAP; Johne’s disease).

In previous work we found that fecal headspace VOCs could be used discriminate between non-vaccinated and Bacillus Calmette-Guerin (BCG) vaccinated white-tailed deer prior to and after virulent *M*. *bovis* challenge with a high degree of SN and SP [38]. The goal of this study was to demonstrate that fecal headspace VOCs could be used to differentiate between non-vaccinated and BCG-vaccinated cattle prior to and post- virulent *M*. *bovis* challenge. Based upon the results of our analysis, we were successful in achieving this goal. The results of this study, combined with our previous studies [30, 32, 38, 39] provide evidence that breath and fecal VOC analysis have merit for disease surveillance in ruminant species. Our work provides data that adds to existing studies in this area, and encourages future development of VOC analyses for disease diagnosis in human and veterinary medical fields.

## Animals, materials and methods

### Ethics statement

All studies were approved by the Institutional Biosafety and Animal Care and Use Committees (IACUC) of the of the United States Department of Agriculture (USDA), Agricultural Research Service (ARS), National Animal Disease Center (NADC), Ames, Iowa, USA (Animal Care and Use Protocol (ACUP) 2688); the USDA, Animal and Plant Health Inspection Service (APHIS), National Wildlife Research Center (NWRC), Fort Collins, Colorado, USA (Protocol QA-2262); and Iowa State University (ISU), Ames, Iowa, USA (IACUC 4-14-7787-B), and were performed within the conditions of the Animal Welfare Act. All animals were housed in appropriate biological containment facilities at NADC under the direct supervision of the institutional clinical veterinarian. Animals were monitored twice daily for overall body condition and well-being, and for any signs of signs of suffering and distress. Strict biosafety protocols were followed to protect personnel from exposure to *M*. *bovis*, including BSL-3 containment upon initiation of *M*. *bovis* challenge in animal rooms and standard BSL-3 and BSL-2 laboratory practices for handling *M*. *bovis* cultures and samples from *M*. *bovis*-infected animals.

### Animals and *Mycobacterium bovis* challenge

One to two day old Holstein bull calves (n = 20) were acquired from a *M*. *bovis*-free, *M*. *avium paratuberculosis*-monitored herd, and placed into a NADC BSL-3 agricultural facility for the duration of the study. Cattle were housed under environmental conditions appropriate for normal physiological thermoregulation; in separate biocontainment rooms; with no exchange of air, food, or water occurring between rooms. Diet consisted of alfalfa hay cubes and a commercial pelleted ration provided twice daily, and water *ad libitum*. All calves were castrated at 42–43 days of age (S1 Table). One individual in the BCG treatment group was humanely euthanized prior to the completion of the study due to a non-study related health issue, decreasing the number of individuals in that cohort (n = 9). Given the low dose challenge and relatively short duration of the study, cattle did not develop clinical signs of bTB (cough, dyspnea, anorexia, weight loss) necessitating palliative therapy.

*Mycobacterium bovis* strain 10–7428_CO_Dairy_10-A (*M*. *bovis* strain 10–7428; USDA, APHIS designation) was used for the challenge inoculum. The inoculum was prepared using standard procedures in Middlebrook 7H9 liquid media (Becton Dickinson, Franklin Lakes, NJ, US) as described by Larsen et al 2005 [40]. Enumeration of *M*. *bovis* challenge inoculum was performed as previously described [41]. Calves were challenged by aerosol with *M*. *bovis* using the method described by Palmer et al 2002 [41] at 120–121 days of age.

### Diagnostic tests performed

Blood samples were collected from all calves seven days prior to vaccination, 20, 42, and 56 days post-vaccination, nine, 16, and 70 days post-challenge for *in vitro* evaluation of cellular immune responses (CMI) to mycobacterial antigens. The CCT was performed according to USDA, APHIS circular 91-45-01 guidelines [42] on all animals 104 days post-challenge (7 days prior to necropsy). Balanced purified protein derivatives (PPDs) were obtained from the Brucella and Mycobacterial Reagents Section of The USDA National Veterinary Services Laboratory, Ames, IA. Calves were administered 0.1 mL (100 μg) *M*. *bovis* PPD (PPDb) and 0.1 mL (40 μg) *M*. *avium* PPD (PPDa) intradermally at separate clipped sites on the side of the neck. Calves were humanely euthanized by intravenous administration of sodium pentobarbital 111 days post-challenge. Necropsy procedures, and gross and microscopic assessment of lesions were performed as described in Palmer et al 2002 [41]. When microscopic lesions consistent with bTB were identified, adjacent sections were stained by the Ziehl Neelsen technique to visualize acid-fast bacilli. Mediastinal, tracheobronchial, medial retropharyngeal, and prefemoral lymph nodes and lungs were sampled for *M*. *bovis* isolation and identification as previously described [43]. Qualitative assessment of mycobacterial colonization was performed using standard mycobacterial culture techniques [44] with Middlebrook 7H11 selective agar plates (Becton Dickinson, Franklin Lakes, NJ, USA) incubated for 8 weeks at 37°C. Confirmation of colonies was performed as previously described [45] using IS6110 real time PCR (RT-PCR).

### Fecal sample collection

Fecal samples were collected per rectum from all calves one day prior to virulent *M*. *bovis* challenge and prior to necropsy. Samples were placed in air-tight 50 mL conical centrifuge tubes, and stored on ice until transport to the NADC Tuberculosis Research Laboratory. Fecal sample processing was performed in a Biosafety Class II cabinet (BSC-II). A 1.5 gm fecal aliquot was removed from each sample using a 3.0 mL hypodermic syringe modified by removing the apex of the syringe barrel. Feces were deposited in the bottom of a clean 20 mL amber glass vial (Wheaton, Millville, NJ, USA) and the vial sealed with an air-tight lid containing a PTFE-lined silicone septum. The outer surface of each vial was decontaminated with tuberculocidal disinfectant (Wexcide, Wexford Labs, Kirkwood, MO USA) and transported in a sealed transfer container to the ISU Atmospheric Air Quality Laboratory for analysis.

### Sample preparation for GC/MS analysis and GC/MS conditions

Pre-concentration of VOCs was performed using a 2 cm 50/30 μm DVB/Carboxen/PDMS (57348-U, Supelco, Bellefonte, PA, USA) SPME fiber mounted in a CTC Combi PAL^™^ LEAP GC autosampler (LEAP Technologies, part of Trajan Family, Inc., Carrboro, NC, USA) equipped with a heated agitator (37°C, 250 rpm agitation). Each sample vial was transferred to the heated agitator, thermally equilibrated for 10 minutes, followed by SPME insertion and headspace exposure for 45 min. The SPME fiber was then transferred and inserted into the GC inlet and desorbed with the GC inlet configured in splitless mode at 250°C using helium (He) carrier gas (99.995% purity). Multi-Dimensional Gas Chromatography–Mass Spectrometry (MDGC-MS) analysis was performed on an Agilent 6890 GC with a restrictor guard column, non-polar capillary column (BP-5, 30.0 m x 530 μm inner diameter x 0.5 μm thickness, SGE, Austin, TX, USA) and polar capillary column (BP-20, 30.0 m x 530 μm inner diameter x 0.5 μm thickness, SGE, Austin, TX, USA) connected in series. Sample flow was split 3:1 via open split interface to an olfactometry port (not used in this research) and mass spectrometer, respectively. Flow to the mass spectrometer was determined by the fixed restrictor column inner diameter and excess effluent was directed to the olfactory sniff port. The carrier gas was set to constant pressure of 5.7 psi at the midpoint (connection point of the non-polar and polar column). Oven temperature was programmed as follows: 40°C for 3.00 minutes, ramped to 240°C at a rate of 7°C/min, and held for 8.43 min. Transfer line to the MS was set at 280°C, and heated zones were 150°C for the single quadrupole and 230°C for the source. The MS was operated in electron ionization (EI) mode, with the electron energy set to 70eV, scanning from *m/z* 34.0–350.0.

### Data analysis

Files containing GC-MS data for two BCG-vaccinated animals were corrupted, resulting in exclusion of these animals from our analysis. Remaining BCG-vaccinated animals (n = 7) were placed into two samples groups based on post-challenge responses to challenge (Table 1). To keep sample group sizes similar for analyses, four non-vaccinated animals were randomly selected for our analyses. Final sample group designations included BCG-vaccinated virulent *M*. *bovis* positive calves (BCGprePOS, BCGpostPOS; n = 3); BCG-vaccinated calves with no evidence of virulent *M*. *bovis* infection (BCGpreNEG, BCGpostNEG; n—4); and non-vaccinated virulent *M*. *bovis* positive calves (NVpre, NVpost; n = 4).

**Table 1:** Sample group designation and diagnostic test results for calves.

Animal ID	Post-challenge Diagnostic Test Results						
	Cfu/gm TBLN virulent *M*. *bovis*^1^	Gross lesion noted in lung	Gross lesion noted in lymph node(s)	Histopathology	ΔPPDb^2^	RT-PCR	Culture
**Non-Vaccinated virulent *M*. *bovis* positive calves**							
NVpre5 NVpost5	2989	+	+	+	28	+	+
NVpre88 NVpost88	1600	+	+	+	62.5	+	+
NVpre91 NVpost91	545	+	+	+	64.5	+	+
NVpre95 NVpost95	82796	+	+	+	43.5	+	+
**BCG-vaccinated virulent *M*. *bovis* positive calves**							
BCGprePOS8 BCGpostPOS8	0	-	+	+	54	+	+
BCGprePOS84 BCGpostPOS84	1058	-	-	+	33.5	+	+
BCGprePOS86 BCGpostPOS86	0	-	-	+	59	+	+
**BCG-vaccinated virulent *M*. *bovis* negative calves**							
BCGpre6 BCGpost6	0	-	-	-	9.5	-	-
BCGpre76 BCGpost76	0	-	-	-	33.5	-	-
BCGpre77 BCGpost77	0	-	-	-	16	-	-
BCGpre85 BCGpost85	0	-	-	-	20.5	-	-

^1^ Colony forming units *M*. *bovis* cultured per gram tracheobronchial lymph node

^2^ Change in response to PPDb pre- vs. post virulent *M*. *bovis* challenge

Paired fecal samples collected from non-vaccinated and BCG-vaccinated calves selected for gas chromatography–mass spectrometry (GC-MS) analysis based on results of diagnostic testing, necropsy, and histopathological results. Samples were collected pre- and post- virulent *M*. *bovis* challenge. Each paired sample is identified by vaccination status, pre- or post-challenge time-point, and infection status (example: non-vaccinated pre-challenge = NVpre; BCG-vaccinated pre-challenge (BCGpre); non-vaccinated post-challenge (NVpost); BCG-vaccinated post-challenge not infected (BCGpostNEG); BCG-vaccinated post-challenge infected (BCGpostPOS).

Baseline-corrected chromatograms were analyzed using the multi-group comparison feature in XCMS Online [46, 47] (www.xcmsonline.scripps.edu) to identify peak ion abundances that differed across sample groups. All remaining statistical analysis were performed using statistical packages available in “R” [48–50]. A principal component analysis (PCA) was performed on the statistically significant ions (α = 0.05, 1.5 fold difference), and the PC scores proportion of variance and cumulative proportion of variance were used to develop five class linear discriminant analysis (LDA) classification models. Training and test datasets containing 70% and 30% of the data were used to produce predicted classifications for each sample, and the results were compared to actual sample group assignment. Misclassification rates were calculated to evaluate the feasibility of using statistically significant ions to discriminate between the sample groups. The number of true positive (infected calves; NVpost, BCGpostPOS); true negative (pre-challenge treatment groups and the post-challenge non-infected BCG-vaccinated calves; NVpre, BCGpre, BCGpostNEG); false positive (true negative calves incorrectly classified as infected), and false negative (true positives incorrectly classified as non-infected) animals were summed. Sensitivity was calculated as the total number of true positives divided by the sum of the true positives plus false negatives, and SP was calculated as the total number of true negative samples divided by the sum of the true negatives plus false positives [51]. An agglomerative hierarchical cluster analysis using Ward’s method was performed to further assess use of ion data as a means to discriminate between individual and grouped samples, and to test the robustness of the PCA and LDA classification models.

Statistically significant ion intensities identified by XCMS Online were retention time matched with total ion chromatographic (TIC) peaks using Agilent Mass Hunter software (Agilent Technologies, Santa Clara, CA, USA), and associated peak areas were incorporated into PCA and LDA analyses to assess the feasibility of using the identified peaks to discriminate between sample groups. Group mean peaks areas were calculated for each peak, and between groups mean peak area fold differences > = 3.0 and biological relevance were used to identify a suite of peaks that best allowed discrimination between the sample groups. This suite of selected peaks was reanalyzed to determine if a minimum number of peaks might be used to discriminate between the sample groups, and whether any peaks might function as biomarkers associated with disease and/or vaccination status. Peaks meeting a minimum spectral match probability > = 65% were tentatively identified using the National Institute of Standards and Technology (NIST) W8N08 database (www.nist.gov), the Kyoto Encyclopedia of Genes and Genomes database [52, 53], and the Human Metabolome Database [54, 55]. When multiple compounds were tentatively identified at one retention time, all tentative compound identifications were considered based on the possibility of co-elution and likelihood that no reference library contains all known compounds.

## Results

### Diagnostic tests

Over the course of the study, no differences in overall health or clinical disease severity were observed among non-vaccinated or BCG-vaccinated calves prior to or after challenge with virulent *M*. *bovis* (excluding the BCG vaccinated animal euthanized prior to study completion). Results of the CCT skin test and *in vitro* evaluation of cellular immune responses to mycobacterial antigens are published elsewhere [56]. Briefly, all calves were classified as reactors based upon standard interpretation of the CCT skin test [42]. Mean responses of NVpost calves to PPDb were significantly greater than responses observed in the BCGpostNEG and BCGpostPOS calves (S1 Fig). Observed responses to PPDb were significantly greater than the responses to PPDa (S2 Fig), and robust cell-mediated immune responses to mycobacterial antigens were present all calves in all treatment groups [56]. Semi-quantitative scoring of gross lesions in the lungs and lymph nodes (tracheobronchial and mediastinal; TBLN), histopathology, and isolation and identification of mycobacterial isolates were performed at NADC (Table 1) [56]. Gross lesions were noted in the lungs and tracheobronchial and mediastinal lymph nodes of all NVpost calves. Gross lesions were noted in the TBLN of one post-challenge BCG-vaccinated calf (BCGpostPOS8). Granulomas containing acid-fast bacteria were identified histologically in the TBLN of all NVpost and BCGpostPOS calves. Calves in the NVpost and BCGpostPOS sample groups were confirmed positive for virulent *M*. *bovis* by culture and RT-PCR. There was no evidence of virulent *M*. *bovis* presence in BCGpostNEG calves.

### Analysis of ion features

**Fig 1:**
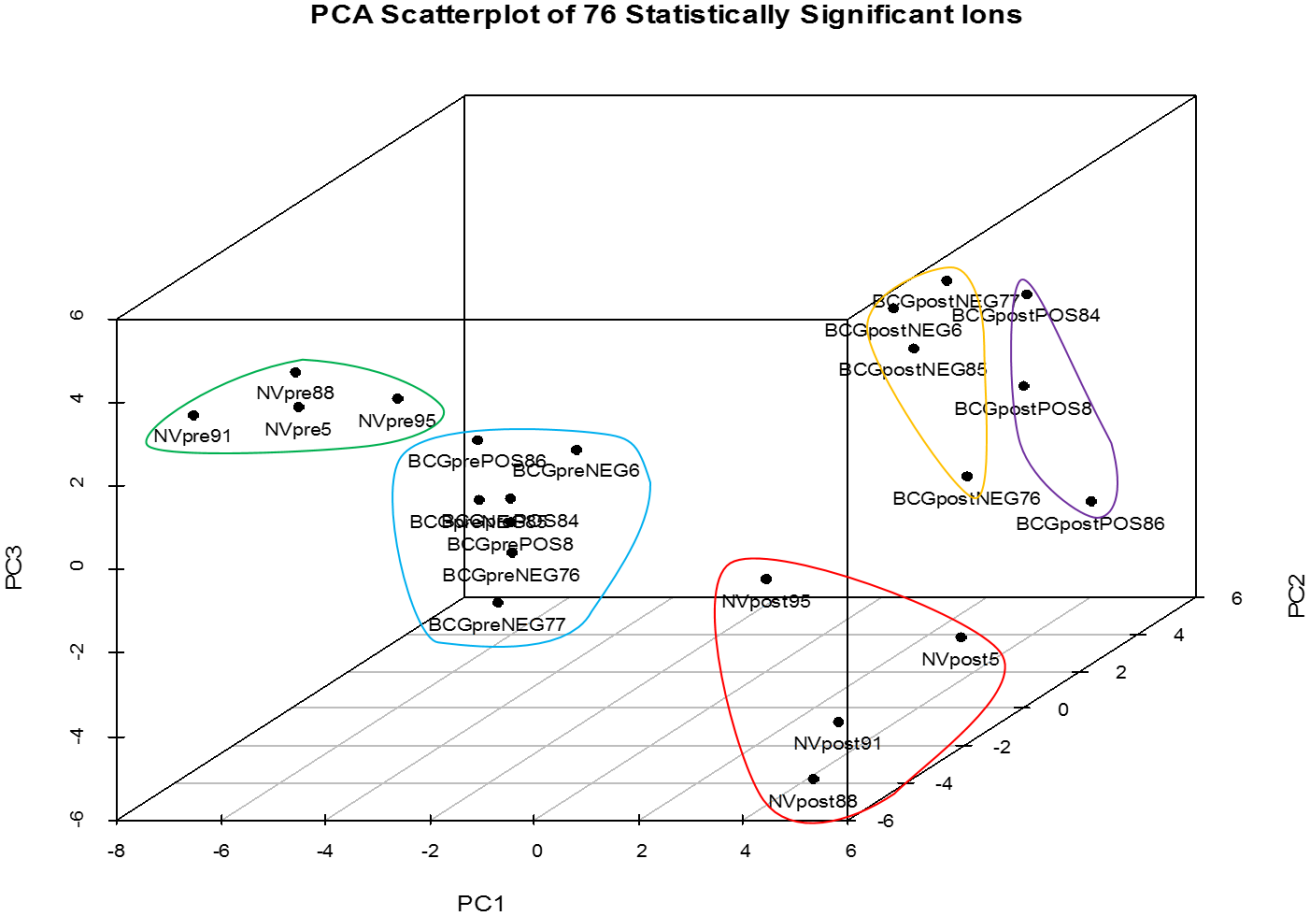
Principal component analysis three dimensional scatterplot of 76 ions. Pre-challenge non-vaccinated (NVpre) and BCG-vaccinated (BCGpreNEG; BCGprePOS) sample groups are indicated by green and blue circles, respectively. Post-challenge virulent *M*. *bovis* positive non-vaccinated (NVpost) and BCG-vaccinated (BCGpostPOS), and virulent *M*. *bovis* negative BCG-vaccinated (BCGpostNEG) sample groups are indicated by red, orange, and purple circles, respectively. Pre- and post-challenge sample groups are separate within the three dimensional space, indicating that pre-challenge and post-challenge calves are different. Pre-challenge sample groups (NVpre; combined BCGprePOS, BCGpreNEG) are separate indicating that these sample groups are distinctly different from each other. Post-challenge sample groups are separate and distinct, indicating that these sample groups are different. Within each sample group, individual samples appear closely associated with each other indicating that intra-group samples are similar to each other.

The analysis performed using XCMS Online identified 180 ions, with 105 ions meeting the criteria for statistical significance (α = 0.05; fold change ≥ 1.5). Visualization of the characteristics of the statistically significant ion features are presented as a cloud plot (S3 Fig) [57]. The ion PCA classification model was developed using 76 of the 105 statistically significant ions. Twenty-nine ions occurring at late retention times (> 34.0 min) attributed to baseline noise were excluded from the analysis. Results are depicted in a three dimensional (3D) scatterplot (Fig 1). Pre- and post-challenge samples groups are distinctly separate within the three dimensional space. Separation is present between NVpre and all BCG-vaccinated (BCGprePOS, BCGpreNEG) sample groups. NVpost, BCGpostPOS and BCGpostNEG sample groups are distinctly separate as well.

Five class LDA classification models were constructed using two through six PC scores. The model constructed using two PC scores resulted in correct classification of 95% of calves (Table 2). All misclassifications consisted of NVpre calves incorrectly classified as BCGpre calves (i.e., true negatives classed into a different true negative group) which did not adversely affect effect SN:SP (100%; 100%). Classification models constructed using three through six PC scores contained no misclassifications, and calculated SN:SPs of 100%:100%.

**Table 2:** Linear discriminant analysis classification of calves using statistically significant ions.

**Number of Principal Components**	**2**	**3**	**4**	**5**	**6**
**Misclassification Rate (%)**	5	0	0	0	0
**Correct Classification Rate (%)**	95	100	100	100	100
**Misclassification**					
• **False Negative (%)**	0	0	0	0	0
∘ Nvpost misclassified Nvpre	0	0	0	0	0
∘ Nvpost misclassified BCGpre	0	0	0	0	0
∘ Nvpost misclassified BCGpostNEG	0	0	0	0	0
∘ BCGpostPOS misclassified Nvpre	0	0	0	0	0
∘ BCGpostPOS misclassified BCGpre	0	0	0	0	0
∘ BCGpostPOS misclassified BCGpostNEG	0	0	0	0	0
• **False Positive (%)**	0	0	0	0	0
∘ Nvpre misclassified Nvpost	0	0	0	0	0
∘ Nvpre misclassified BCGpost POS	0	0	0	0	0
∘ BCGpre misclassified Nvpost	0	0	0	0	0
∘ BCGpre misclassified BCGpostPOS	0	0	0	0	0
∘ BCGpostNEG misclassified BCGpostPOS	0	0	0	0	0
∘ BCGpostNEG misclassified NVpost	0	0	0	0	0
• **True Negative (%)**	0	0	0	0	0
∘ Nvpre misclassified BCGpre	100	0	0	0	0
∘ Nvpre misclassified BCGpostNEG	0	0	0	0	0
∘ BCGpre misclassified Nvpre	0	0	0	0	0
∘ BCGpre misclassified BCGpostNEG	0	0	0	0	0
∘ BCGpostNEG misclassified Nvpre	0	0	0	0	0
∘ BCGpostNEG misclassified BCGpre	0	0	0	0	0
• **True Positive (%)**	0	0	0	0	0
∘ Nvpost misclassified BCGpostPOS	0	0	0	0	0
∘ BCGpostPOS misclassified Nvpost	0	0	0	0	0
**Sensitivity (%)**	**100**	**100**	**100**	**100**	**100**
**Specificity (%)**	**100**	**100**	**100**	**100**	**100**

Five class models were constructed using two through six principal component analysis (PCA) dimensional scores. Models resulting in optimal misclassifications (0%), and SN:SP (100%:100%) were constructed using three through six PC scores. The model constructed using two PC scores returned the highest misclassification rate (5%); however, all misclassifications (100%) consisted of NVpre (true negative) animals misclassified as BCGpreNEG animals (true negatives), which did not affect the SN:SP (100%:100%).

**Fig 2:**
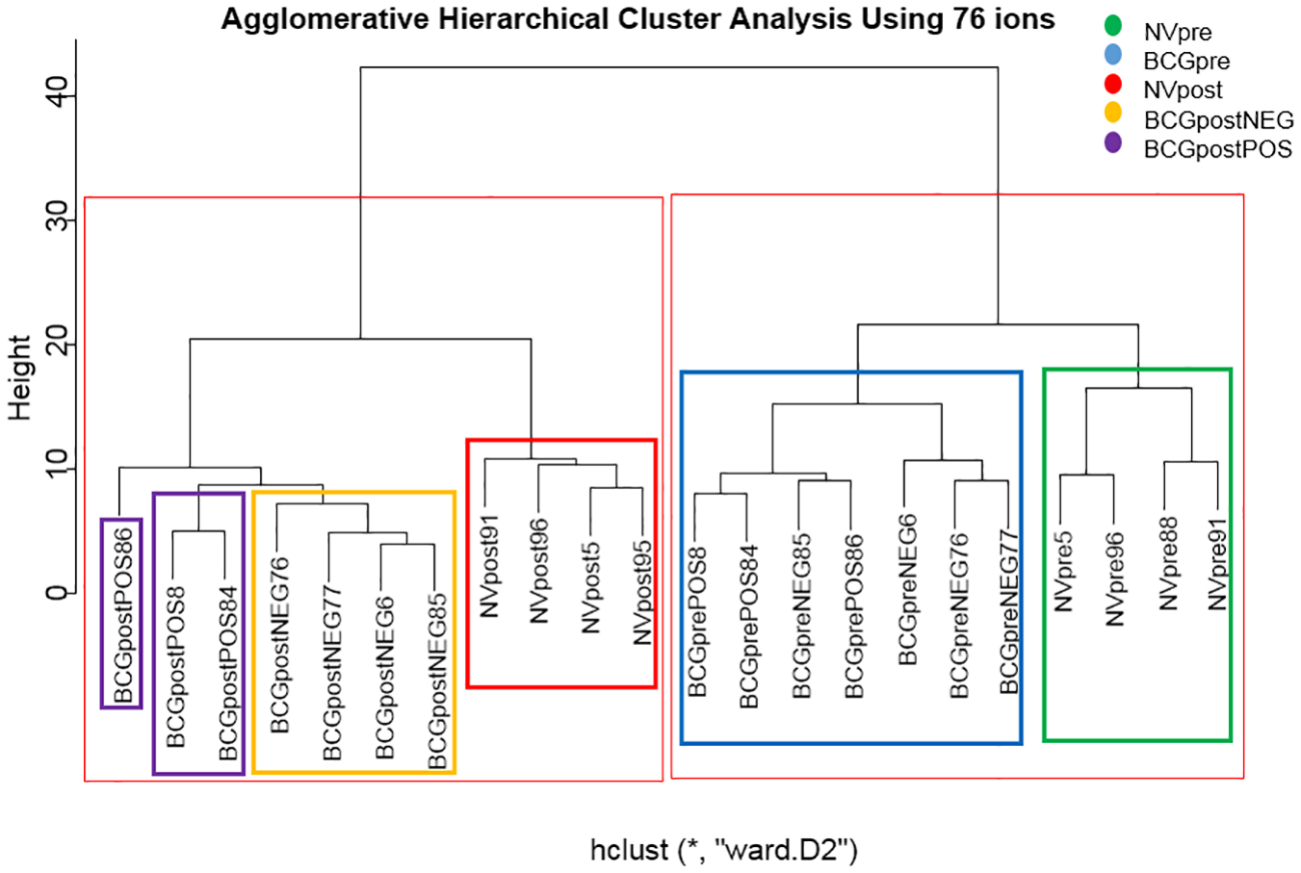
Agglomerative hierarchical cluster analysis constructed using 76 ions. Initial pairing of individuals of respective sample groups is present. Progression through the hierarchy places paired individuals into respective sample groups. The final separation places each individual its respective pre- or post-challenge group. Clear separation of pre- and post-challenge sample groups is observed. Clear separation of NVpre and BCGpre individuals indicates that these animals are distinctly different. All BCGpreNEG and BCGprePOS samples are within one cluster, indicating similarity prior to virulent *M*. *bovis* challenge. Post-challenge BCGpostNEG and BCGpostPOS samples within separate, but closely associated clusters, indicates that while difference among those individuals is present, they still are similar. All NVpostPOS individuals are found in a distinct cluster indicating a strong inter-group variation.

The ion cluster analysis shows initial pairing of individual calves within respective sample groups. Subsequent progression places paired individuals into respective pre- and post- virulent *M*. *bovis* challenge groups (Fig 2). The final clustering produces clear separation between pre- and post-challenge sample groups. Inter-group discrimination is present between NVpre and BCGpre animals. All BCGpreNEG and BCGprePOS samples are found within one cluster, indicating that these samples are similar prior to virulent *M*. *bovis* challenge. After challenge, BCGpostNEG and BCGpostPOS samples are found within separate, but closely associated clusters, indicating that while the presence of virulent *M*. *bovis* in BCGpostPOS animals has induced some difference in those individuals, they still are similar. The location of all NVpostPOS individuals into a distinct cluster indicates that lack of BCG-vaccination prior to challenge creates a strong inter-group variation.

#### Analysis of peak areas and compound identification

Ions identified as statistically significant were GC column retention time matched to 23 chromatographic peaks. Ten peaks were identified by single ion matches, while the remainder (n = 13) were matched to multiple ions. Peak area data were visually inspected for biological relevance, and using these criteria, eight peaks were excluded from consideration. Two additional peaks identified as silane-containing compounds were removed because they were likely contaminants eluted from the GC column or septa material, leaving 13 peaks available for further analysis and tentative compound identification.

**Fig 3:**
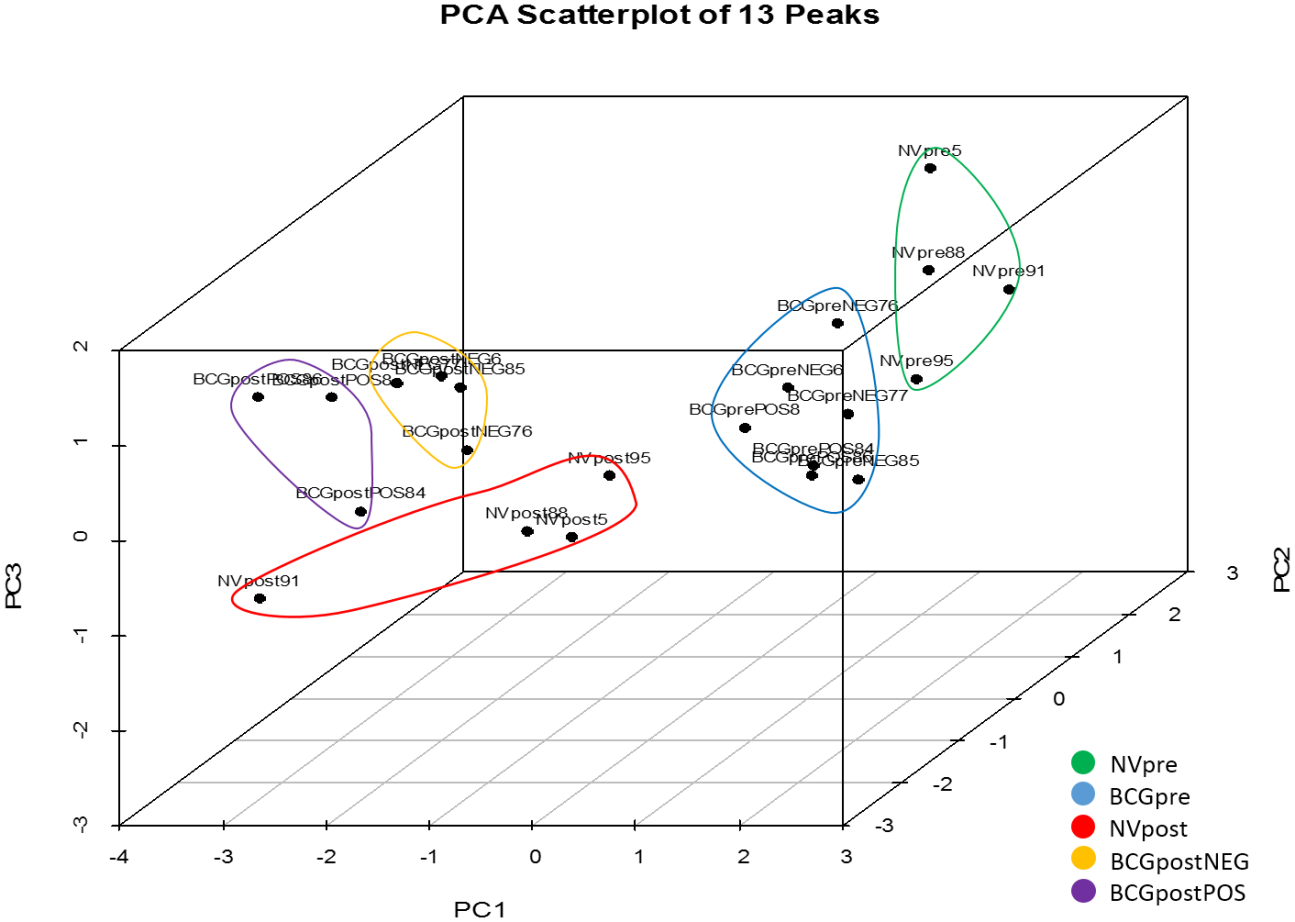
Three dimensional principal components analysis scatterplot of 13 peaks. All BCG-vaccinated individuals are located within one cluster prior to challenge indicating that there is little difference present among vaccinated individuals at this time-point. NVpre individuals are located in a cluster distinctly separate from BCGpre individuals. Pre- and post-challenge sample groups are separate, indicating that differences exist between pre-and post-challenge individuals. Post-challenge, two BCG sample groups are present, and are distinctly separate from the NVpost sample group. Individuals within treatment groups cluster in close association indicating similarity among individuals with respect to vaccination and/or persistence of virulent *M*. *bovis*.

The peak area PCA classification analysis was reliably capable of discriminating between the sample groups (Fig 3). Separation of pre- and post-challenge samples is present indicating that exposure to virulent *M*. *bovis* induces distinct changes in all individuals regardless of vaccination status. Individuals within treatment groups cluster in close association indicating similarity. BCGpreNEG and BCGprePOS animals cluster together indicating that there is little difference between BCG-vaccinated individuals prior to virulent *M*. *bovis* challenge. The BCGpre sample group and NVpre sample groups are separate. Post-challenge the BCGpostNEG and BCGpostPOS groups form separate clusters, indicating that the persistent presence of virulent *M*. *bovis* in BCGpostPOS animals induces differences between these groups. NVpost individuals form a separate cluster with the exception of one pre-challenge non-vaccinated animal (NVpost91).

LDA classification models were developed using two through four PCs. Models returning the lowest misclassification rates were constructed using two and four PC scores (Table 3). No misclassifications were observed, and corresponding SN:SP were 100%:100%, respectively. The misclassification rate for the model constructed using three PC scores was 17%, with all misclassified samples (100%) consisting of BCGpostPOS calves misclassified as BCGpostNEG (false negative samples). The calculated SN:SP for this model were 83%:100%.

**Table 3:** Five way linear discriminant analysis classification of calves using 13 peaks.

**Number of Principal Components**	**2**	**3**	**4**
**Misclassification Rate (%)**	0	17	0
**Correct Classification Rate (%)**	100	83	100
**Misclassification**			
• **False Negative (%)**	0	0	0
∘ Nvpost misclassified Nvpre	0	0	0
∘ Nvpost misclassified BCGpre	0	0	0
∘ Nvpost misclassified BCGpostNEG	0	0	0
∘ BCGpostPOS misclassified Nvpre	0	0	0
∘ BCGpostPOS misclassified BCGpre	0	0	0
∘ BCGpostPOS misclassified BCGpostNEG	0	100	0
• **False Positive (%)**	0	0	0
∘ Nvpre misclassified Nvpost	0	0	0
∘ Nvpre misclassified BCGpost POS	0	0	0
∘ BCGpre misclassified Nvpost	0	0	0
∘ BCGpre misclassified BCGpostPOS	0	0	0
∘ BCGpostNEG misclassified BCGpostPOS	0	0	0
∘ BCGpostNEG misclassified NVpost	0	0	0
• **True Negative (%)**	0	0	0
∘ Nvpre misclassified BCGpre	0	0	0
∘ Nvpre misclassified BCGpostNEG	0	0	0
∘ BCGpre misclassified Nvpre	0	0	0
∘ BCGpre misclassified BCGpostNEG	0	0	0
∘ BCGpostNEG misclassified Nvpre	0	0	0
∘ BCGpostNEG misclassified BCGpre	0	0	0
• **True Positive (%)**	0	0	0
∘ Nvpost misclassified BCGpostPOS	0	0	0
∘ BCGpostPOS misclassified Nvpost	0	0	0
**Sensitivity (%)**	**100**	**83**	**100**
**Specificity (%)**	**100**	**100**	**100**

Models resulting in lowest misclassification (0%) and optimal SN:SP (100%:100%) were constructed using two and four PC scores. The model developed using three PC scores returned a misclassification rate of 83%. All misclassifications occurring (100%) in this model were false negatives (BCGpostPOS animals misclassified BCGpostNEG).

**Fig 4:**
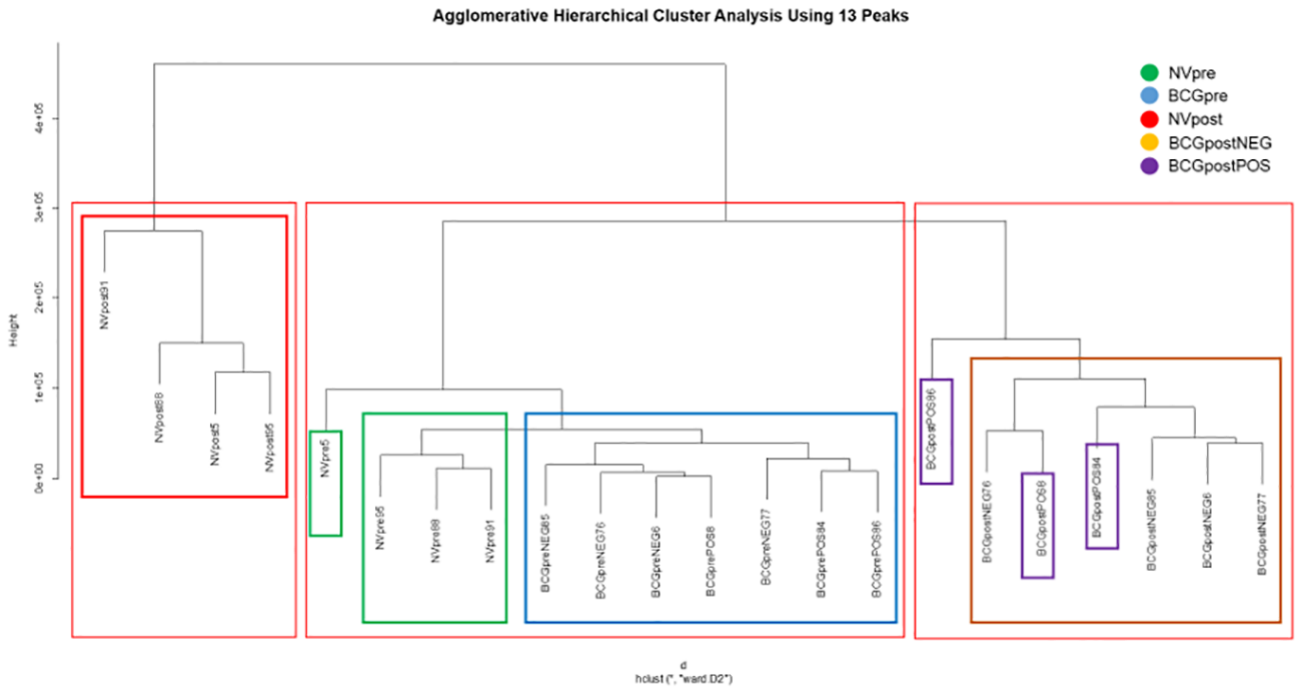
Agglomerative hierarchical cluster analysis dendrogram constructed using 13 peaks. Initial pairings resulted in grouping of like samples. In the next hierarchical grouping, three NVpre samples (NVpre88, 91 and 95) appear more closely associated with the BCGpre sample group than NVpre5, and two BCGpostPOS (BCGpostPOS4 and 8) samples group with the BCGpostNEG samples. The final hierarchical division identifies the NVpost samples as a distinct group separate from the grouped pre-challenge samples, and the post-challenge BCG-vaccinated samples.

The peak area cluster analysis shows pairing of individuals within treatment group designation (Fig 4). As the hierarchical model progresses, two BCGpostPOS animals (BCGpostPOS84, BCGpostPOS86) group among the BCGpostNEG animals. The remaining BCGpostPOS animal occupies a separate space in the tree. In the next hierarchical progression, three groups are identified. NVpost animals form a distinct group; all pre-challenge animals (NVpre; BCGpre) fall within a second group; and all post-challenge BCG-vaccinated animals form a third group. The final two groupings consist of one cluster containing all NVpost animals, and another containing all the other animals, indicating that the NVpost cattle are truly unique compared to the other sampled populations. Within the group containing all other treatment groups, NVpre and BCGpre sample groups are distinct from the BCGpostNEG and BCGpostPOS groups. The two misclassified BCGpostPOS animals (BCGpostPOS84, BCGpostPOS86) are identical to those identified in the ion cluster analysis.

Tentative identifications of peaks and associated information are summarized in Table 4. Briefly, compounds tentatively identified using a *>* = 65% probability match in the NISTW8N08 library include an alcohol; an aldehyde; an alkane; an imine; an indole; one ketone; a pyridine derivative; and a thioether. Five peaks could not be identified using this database. Compounds tentatively identified using the KEGG and HMDB databases that differed from NIST included an organosulfur; an amino acid; an alcohol; a salt of chloric acid; a fatty acid; and a dicarboxylic acid. Five peaks were not identified by KEGG or HMDB. Three compounds could not be identified by any of the databases used.

**Table 4:** Tentative compound identification of chromatographic peaks.

Peak	Retention Time	Tentative Compound Identification	Database Identification Number	Compound Class
		Significant Ions		
1	3.32	Dimethyl sulfide	NIST W8N08	Thioether
		62(999) 47(953) 45(408) 46(361) 61(333)	HMDB02303	
			KEGG00580	
		3-methylthiophene	HMDB33119	Thiophene
		97(999) 98(530) 45(174) 53(88) 39(81)		
2	15.60	Benzaldehyde	NIST W8N08	Aldehyde
		77(999) 106(935) 105(889) 51(495) 50(296)	KEGG 00261	
			HMDB0611	
		Dimethyl sulfone (methylsulfonylmethane, MSM)	KEGG11142	Organosulfur (sulfone)
		79(999) 15(830) 94(477) 81(49) 43(45)	HMDB04983	
3	16.02	Unknown		
4	18.64	Methoxy-phenyl-oxime	NIST W8N08	Imine
		133(999) 151(649) 135(262) 73(167) 42(165)		
5	18.81	2-Methylpyridine (2-picoline)	NIST W8N08	Pyridine derivative
		93(999) 66(520) 92(288) 65(212) 78(206)		
6	18.94	Citrulline	KEGGC00327	Amino acid
		69(999) 43(663) 56(638) 30(570) 28(459)	HMDB00904	
7	19.14	Unknown		
8	25.31	Unknown		
9	28.19	2-dodecanone	NISTW8N08	Ketone
		58(999) 43(752) 59(308) 71(289) 49(169)	HMDB31019	
		2-chloro-1-propanol	HMDB31335	Alcohol
		31(999) 58(270) 27(177) 29(151) 62(132)		
10	28.61	Chlorate	KEGG01485	Inorganic non-metallic compounds containing a chlorate as the largest oxoanion
			HMDB02036	
11	28.68	1H-Indole	NISTW8N08	Indole
			HMDB00738	
			KEGG00463	
12	29.44	2, 6, 10, 14-tetramethylhexadecane (phytane)	NIST W8N08	Diterpenoid alkane
		57(999) 71(712) 43(612) 85(349) 41(313)		
		5-hydroxy-lysine	KEGG16741	Fatty acyl (amino acid derivative
		70(999) 82(830) 43(684) 42(473) 56(469)	HMDB06827	
13	30.13	3, 7, 11, 15-tetramethyl-2-hexadecene1-ol (phytol)	NISTW8N08	Diterpene alcohol
		71(999) 43(361) 57(333) 55(299) 69(278)	HMDB02019	
			KEGGC01389	
		Succinic acid (butanedoic acid	KEGG00042	Dicarboxylic acid and derivative
		55(999) 45(842) 74(753) 27(636) 100(606)	HMDB00254	

Chromatographic peaks were tentatively identified using three databases. Statistically significant ions identified by XCMS were included in identification of compounds. The first five significant ion fragments as identified by compound matching are noted for reference.

Fold changes in mean peak area between treatment groups were calculated to identify peaks with greatest potential to perform dependent (pre- *vs*. post-challenge) and independent (vaccination and infection status) groups comparisons (S2 Table). A mean fold difference > = 3.0 was used to identify peaks that best allowed inter- and intra-groups discrimination. The results of this comparison demonstrate that while specific fecal VOC biomarkers indicative of vaccination and/or disease status were not identified, differentiation between groups is still possible using suites of VOCs (Table 5). In the NVpre *vs*. NVpost comparison, twelve peaks had mean peak area fold differences meeting our selection criteria, with six peaks (2–4, 7, 11–12) having the greatest differences. For the BCGpreNEG *vs*. BCGpostNEG comparison, 10 peaks were identified, with 7 peaks (1–5, 9, 10) providing best discrimination. Six peaks (1–5 and 10) out of eleven provide best differentiation between BCGprePOS and BCGpostPOS animals. NVpre and BCGpre animals are discriminated by one peak (peak 7). For the NVpre *vs*. BCGpostNEG groups comparison a suite of 11 peaks (1–10, 13) met our selection criteria, and four peaks (1–3, 7) had the greatest mean fold differences. Four of 11 peaks (1–3 and 7) allow best discrimination between NVpre and BCGpostPOS samples. Discrimination between NVpost and BCGpre animals is best achieved using five (2–4, 11, 12) of 11 peaks. Four (peaks 8, 10–12) of eight peaks are best capable of discriminating between NVpost and BCGpostNEG animals. A suite of seven VOCs was identified in the NVpost *vs*. BCGpostPOS comparison, with two peaks (8, 12) having the greatest mean fold difference. Two peaks (peaks 11 and 9) comprise the suite of VOCs best capable of discriminating between BCGpostNEG and BCGpostPOS animals. While we were unable to identify a specific biomarker indicative of vaccination status or presence of virulent *M*. *bovis* infection, the results of our analysis do demonstrate the merit in evaluating multiple VOCs as a means of identifying animal cohorts.

**Table 5:** VOC suites allowing discrimination between sample groups.

Group Comparison	Peaks														
	1	2	3	4	5	6	7	8	9	10	11	12	13	Total Peaks Identified	Peaks with greatest mean fold difference
NVpre vs. NVpost														12	6
BCGpreNEG vs. BCGpostNEG														10	7
BCGprePOS vs. BCGpostPOS														11	6
NVpre vs. BCGpre														1	1
NVpre vs. BCGpostNEG														11	4
NVpre vs. BCGpostPOS														11	4
NVpost vs. BCGpre														10	5
NVpost vs. BCGpostNEG														8	4
NVpost vs. BCGpostPOS														7	2
BCGpostNEG vs. BCGpostPOS														2	2

Peak area mean fold differences were calculated for dependent (pre- vs. post-challenge) and independent (vaccination and infection status) treatment group comparisons. Suites of peaks with fold differences meeting a minimum selection criteria (> = 3.0) were identified for each comparison. Peaks with greatest mean fold differences are denoted in black. Peaks with lower mean fold differences are noted in gray. Peaks that do not meet the selection criteria are denoted in white.

Potential sources, and cellular, biological or metabolic functions associated with each tentatively identified compound were explored using KEGG, HMDB, and literature searches [32, 38, 53–55, 58–77] (Tables 6 and 7). Extensive metabolic information relative to *M*. *bovis* is not published; therefore, we referred to information published for *M*. *tuberculosis* when necessary. Eight tentatively identified compounds could be associated with mammalian systems. Another eight compounds could be associated with microbial cellular function. Seven compounds appear associated with mycobacterial metabolism, seven have been identified in cattle, four appear to be involved in non-mycobacterial microbial metabolism, and two are associated with immunological function. Overlap occurs among multiple compounds. For example, citrulline is associated with macrophage metabolism and function; is produced as by-product of the urea cycle, and is utilized by rumen microflora to convert urea to ammonia.

**Table 6:** Potential sources of peak compounds, and order of sample group mean peak areas.

Peak	Tentative Compound Identification	Tentative Associations [32, 38, 54, 55, 58–77]85, 86	Order of Mean Peak Area Fold Difference
**1**	Dimethyl sulfide	Found in culture headspace of *M*. *bovis* BCG Produced by bacterial metabolism of methanethiol *M*. *tuberculosis* and other mycobacteria possess the mddA gene, which encodes a methyltransferase that generates dimethyl sulfide via methylation of methanethiol	BCGpostPOS = BCGpostNEG > NVpost > NVpre = BCGpre
	3-methylthiophene	Rumen byproduct	
**2**	Benzaldehyde	Found in some plant material Found in feces of humans and animals infected with *Clostridium difficile* or *Campylobacter jejuni*	NVpost > BGGpostPOS = BCGpostNEG > BCGpre > NVpre
	Dimethyl sulfone (methylsulfonylmethane, MSM)	Byproduct of intestinal bacterial metabolism Occurs in some plants Normal concentrations in human plasma and CSF VOC associated with rumen metabolism	
**3**	Unknown		BCGpostPOS = BCGpostNEG > NVpost > BCGpre > NVpre
**4**	Methoxy-phenyl-oxime	Imines are common in nature. Example: Vitamin B6 promotes the deamination of amino acids via the formation of imines	NVpost > BCGpostPOS = BCGpostNEG > BCGpre > NVpre
**5**	2-Methylpyridine (2-picoline)	May have some inhibitory function against mycobacterial pantothenate synthetase	BCGpostPOS > BCGpostNEG = NVpost > BCGpre > NVpre
**6**	Citrulline	Produced by ornithine and carbamoyl phosphate in a central reaction of the urea cycle. Produced as a byproduct during nitric oxide synthesis from arginine. Nitric oxide is synthesized by macrophages from extracellular arginine during early infection, generating citrulline as a byproduct. If extracellular arginine is depleted, reverse synthesis of arginine from citrulline sustains NO production via arginosuccinate synthase. Macrophages lacking this enzyme do not salvage citrulline and are ineffective in controlling mycobacterial infection.	NVpre > BCGpre > NVpost > BCGpostPOS = BCGpostNEG
**7**	Unknown		NVpre > BCGpostPOS > BCGpostNEG = NVpost > BCGpre
**8**	Unknown		NVpost > BCGpostPOS > BCGpostNEG > NVpre = BCGpre
**9**	2-dodecanone	Found in alcoholic beverages Found in rue, hop, and tomato leaf oils. Identified in feces of white-tailed deer. VOC produced by cattle.	NVpre > BCGpre > NVpost > BCGpostPOS > BCGpostNEG
	2-chloro-1-propanol	Used for etherification of food starch	
**10**	Chlorate	Term includes salts of chloric acid. Natural deposits are found in nature. Various microorganisms are capable of reducing chlorate to chloride. Perchlorate reducing bacteria utilize chlorate as a terminal electron acceptor. Selective inhibitor of PAPS, which is required by HEVs in lymphoid tissues which support lymphocyte extravasation from blood. Can be present in food, chlorinated water, or in some disinfectants	BCGpostPOS = BCGpostNEG > NVpost > BCGpre = NVpre
**11**	Indole	VOC produced by cattle. Produced by bacteria as a part of tryptophan metabolism. Regulates various aspects of bacterial physiology such as spore formation, plasmid stability, drug resistance, biofilm formation and virulence. The tryptophan biosynthetic pathway is integral to *M*. *tuberculosis* survival.	NVpost > BCGpostPOS > NVpre = BCGpre = BCGpostNEG
**12**	2, 6, 10, 14-tetramethylhexadecane (phytane)	Derivative of chlorophyll. Found in bovine liver, heart, muscle, fat. Has been described as a compound capable of binding to CYP124, a catabolic P450 enzyme involved in *M*. *tuberculosis* biosynthesis and metabolism.	NVpost > BCGpostPOS > BCGpostNEG > NVpre = BCGpre
	5-hydroxylysine	Amino acid derivative of lysine found in collagenous tissue including bovine conglutinin, a serum protein capable of binding to immune complexes via complement component C3bi. Lysine is converted to hydroxylysine in the biosynthesis of mycobactin, a high density lipid hexdentate iron ligand utilized by *M*. *tuberculosis* to scavenge host non-heme iron for DNA synthesis and respiration.	
**13**	3, 7, 11, 15-tetramethyl-2-hexadecene1-ol (phytol)	Liberated from ruminant gut fermentation of plants, converted to phytanic acid and stored in fats. Decomposition product of chlorophyll. Found in tocopherol (Vitamin E), and phylloquinone (Vitamin K1). Has been observed to act as a ligand and will bind with PKS 18 and AccD5 receptors of *M*. *tuberculosis*. Phytanic acid is bound by *M*. *tuberculosis* CYP124 receptors.	NVpost = BCGpostPOS > BCGpostNEG > BCGpre > NVpre
	Succinic acid (butanedoic acid)	Occurs naturally in plants, animals, soils. Anion (succinate) is a component of the citric acid cycle. Succinate dehydrogenase (SDH) is important in mitochondrial function (part of respiratory chain and Krebs cycle). Produced by fermentation of glucose. Succinic acid producing bacteria have been isolated from the rumen of cattle. *M*. *tuberculosis* utilizes the anion (succinate) to sustain membrane potential, ATP synthesis, and anaplerosis, and can remodel the tricarboxylic acid cycle to increase production of succinate in response to hypoxia. Methyl-nicotinate has been reported as a potential biomarker for M. tuberculosis. Nicotinamide is a compound with known tuberculocidal activity.	

Metabolomics data base and literature searches were utilized to identify potential sources and associations of the VOCs identified. Sample groups are arranged in descending order of mean peak area values to demonstrate how each VOC may be used to identify sample groups.

**Table 7:** Potential sites of origin; cellular and biofluid locations; and biofunctions of tentatively identified volatile organic compounds.

Peak	Tentative Compound Identification	Origin	Cellular Location	Biofluid or Tissue	Biofunction
**1**	Dimethyl sulfide	Endogenous Microbial	Cytoplasm	Blood CSF Feces Urine Fat Intestine Kidney Liver	Osmolyte Enzyme cofactor Signaling Waste products Sulfur metabolism
	3-methylthiophene				
**2**	Benzaldehyd3	Endogenous		Blood Feces Saliva	Toluene degradation
	Dimethyl sulfone (methylsulfonylmethane, MSM)	Endogenous Microbial	Cytoplasm	Blood CSF Urine Saliva	Osmolyte Enzyme cofactor Signaling Sulfur metabolism Waste product Methanethiol metabolism
**3**	Unknown				
**4**	Methoxy-phenyl-oxime				Immines are common in nature. Example: Vitamin B6 promotes the deamination of amino acids via the formation of imines
**5**	2-Methylpyridine (2-picoline)				
**6**	Citrulline	Endogenous	Mitochondria	Blood CSF Saliva Urine Epidermis Fibroblasts GIT Kidney Liver Neural Tissue Placenta Platelet Prostate	Byproduct of Urea Cycle Arginine biosynthesis. Aspartate, alanine and proline metabolism. Biosynthesis of amino acids
**7**	Unknown				
**8**	Unknown				
**9**	2-dodecanone	Endogenous Food	Membrane	Feces Saliva	Nutrient
	2-chloro-1-propanol	Endogenous Food	Cytoplasm Extracellular		Nutrient
**10**	Chlorate	Endogenous			Osmolyte Enzyme cofactor Signaling
**11**	Indole	Endogenous Microbial	Membrane	Epidermis Feces Saliva Urine, Fibroblasts GIT Neural Tissues	Tryptophan metabolism Phenylalanine, tyrosine, tryptophan biosynthesis Protein digestion and absorption
**12**	2, 6, 10, 14-tetramethylhexadecane (phytane)				
	5-hydroxylysine	Endogenous	Cytoplasm	Blood Urine	Protein synthesis Amino acid biosynthesis Lysine degradation
**13**	3, 7, 11, 15-tetramethyl-2-hexadecene1-ol (phytol)	Food	Extracellular Membrane	Fibroblasts	Nutrient Stabilizer Surfactants Emulsifier Cell signaling Fuel and energy storage Fuel or energy source Membrane integrity and stability
	Succinic acid (butanedoic acid)	Endogenous Microbial	Extracellular Mitochondria Endoplasmic reticulum Peroxisome	Blood CSF Feces Saliva Urine Fat Neural Tissue Liver Muscle Pancreas Placenta Spleen	Alanine, proline, butanoate, butyrate, C5-branched dibasic acid, glutamate, propanoate, tyrosine, phenylalanine, pyruvate, propanoate metabolism Valine, leucine, isoleucine degradation DNA component Carnitine synthesis Ketone body metabolism Mitochondrial electron transport chain Citrate cycle Glucagon signaling pathway Succinate dehydrogenase component Nicotinate and nicotinamide metabolism

Metabolomics data base and literature searches were used to identify biological sites of origin, and physiological locations and biofunctions of the tentatively identified VOCs.

## Discussion

We were successful in discriminating between sample groups using statistically significant ion data extracted from our raw data by XCMS Online. The PCA performed on these data resulted in correct classification of all animals to their respective dependent (pre- and post- virulent *M*. *bovis* challenge) and independent (vaccination and infection) groups. The LDA classification models resulted in correct classification rates of 95–100% (0–5% misclassification). The cluster analysis misclassed two BCGpostPOS animals as BCGpostNEG (false negatives). The best use of the ion data was to evaluate the raw data in a preliminary format. For example, in this study, the number of statistically significant ions associated with a specific peak inconsistently ranged from a single ion to six ions, potentially biasing the analysis toward over or under representation of certain chromatographic peaks. Because ion count data do not reflect peak area, ion intensity does not equate to differences in VOCs between individuals or treatment groups. The intensity of a single ion may not represent the parent ion for a compound, and may result from the ionization of multiple co-eluting compounds that share a common ionization fragment. Finally, relative to the intended purpose of our research (assessment of host-pathogen interactions), associated peaks need to be biologically logical in occurrence. This cannot be ascertained using ion data.

The results generated by the peak area classification models are epidemiologically acceptable. The calculated SN:SP of our optimal LDA classification models were 100%:100%, and 83%:100% for the model that performed poorly. In comparison, the SN:SP of the CFT, comparative CCT, and the IFN-ɣ assay have been reported to range from 68–96.8%:96–98%; 55.1–93.5%:88.8–100% [2]; and 73–100%:87.7–99.2% [78], respectively. These results could be adequately visualized in the PCA scatterplot. The cluster analysis misclassified two BCGpostPOS animals as BCGpostNEG (false negatives). An advantage of the cluster analysis was its ability to independently group data, eliminating potential selection bias on the part of the researcher, unlike LDA which requires that the groups be defined prior to analysis. As such, we found the cluster analysis a helpful tool to evaluate the robustness of our other analyses.

We were successful in identifying associations between VOCs and vaccination status and/ or the presence of *M*. *bovis* infection. While individual VOCs did not appear to be exclusively associated with sample cohorts, use of inter- and intra-group mean peak area fold differences did identify suites of VOCs that allowed discrimination between independent and dependent treatment groups. An analogy would be use of a blood chemistry analysis to assess the overall function of all of an animal’s health, followed by evaluation of specific suites of individual blood chemistries to assess renal, hepatic or pancreatic function.

These findings may infer that the changes noted are associated with host immunological function; however, given that the BCG-vaccine is comprised of attenuated *M*. *bovis* that can persist in host tissues [79], and the pathological persistence of virulent *M*. *bovis* in infected hosts, it is plausible that some VOC changes reflect microbial and/or host metabolic, homeostatic, or physiological processes. In this study, five of our tentatively identified compounds have been associated with *M*. *tuberculosis* metabolism. Because reference libraries are not exclusive or complete, and the likelihood that biological samples contain unknown compounds. it is important to consider all potential chemical or metabolic matches [80]. Attempting to identify unknown compounds, or those with a low probability matches using a battery of analytical standards is cost prohibitive and was not performed in this study.

We can state with confidence that our findings demonstrate it is possible to differentiate between non- and BCG-vaccinated Holstein cattle before and after virulent *M*. *bovis* challenge using fecal VOCs collected by methods similar but more refined than those used in a similar study of white-tailed deer [38]. Two compounds identified by both studies (2-dodecanone, 2-methyl pyridine) shared the same trend in peak area fold difference between treatment groups (cattle: NVpost > BCGpostPOS > BCGpostNEG > BCGpre > NVpre; white-tailed deer: NVpost > BCGpostNEG > BCGpre > NVpre, there were no BCGpostPOS deer in this study). A third compound (1H-indole) followed the described sample progression pattern in white-tailed deer, and progressed by treatment group in cattle as NVpost > BCGpostPOS > BCGpostNEG > NVpre = BCGpre, results that are similar and warrant further exploration. None of the compounds found in the suite of breath VOCs used to discriminate between healthy and virulent *M*. *bovis* infected cattle in our pilot study were identified here [32]. Several factors such as the sample (breath *vs*. feces); VOC collection method (sorbent tubes *vs*. SPME); and differences in GCMS analysis methods may have contributed to this disparity. Five identified compounds (1H-indole, 2-methyl pyridine, benzaldehyde, dimethyl sulfide, dimethyl sulfone) have been associated with normal ruminant physiology in cattle [61, 81]. Dimethyl sulfide has been previously reported in the literature as a compound associated with *M*. *bovis* cultures [58]. Eight of our tentatively identified compounds have not been previously reported in our prior studies or elsewhere.

We were not successful in detecting a specific candidate VOC biomarker to indicate BCG vaccination or pathogenic *M*. *bovis* exposure/infection. Tentative biomarkers associated with *M*. *tuberculosis* infection have been suggested [19, 58, 82, 83]; however, because of the influence of diet, behavior, and other factors, identification of one or two compounds as specific indicators of infection by a unique pathogen is difficult. For example, methyl-nicotinate, a compound proposed as a *M*. *tuberculosis* biomarker, can be found in the breath of smokers [84], is used as a flavoring ingredient, and is present in coffee, various nuts, alcoholic beverages, and fruits [54, 55]. Instead, we demonstrate that a suites of VOCs may be used to discriminate between unique treatment groups prior and after challenge with virulent *M*. *bovis*. If the VOCs identified in this study are associated only with non-specific host adaptive or immune responses to pathogen presence, comparative research must be conducted exploring the suites of VOCs produced by other infectious diseases of cattle.

Strengths of our study include analysis of samples from physically similar Holstein steers housed in controlled environmental and husbandry conditions, and exposed to a known concentration of *M*. *bovis* inoculum. Weaknesses include changes in our collection and sample analysis method which were intentional and done with the understanding that retrospective comparison to previous results may be compromised. The small number of samples available for analysis was unavoidable given the expense of housing large ungulates in confinement; however; this study represents the third time we have been able to discriminate between non-infected and *M*. *bovis* infected animals using VOCs present in breath [32] or feces [38]. Repeatability infers reliability, and repeated studies will allow compilation of a library of identified VOCs that will better define suites of VOCs appropriate for identification of disease or vaccination status.

Developing a disease surveillance modality that uses a readily accessible biological sample (feces) collected directly from domestic livestock would improve surveillance strategies by reducing or eliminating animal handling events, decrease animal stress, allow better opportunity for retesting and storage of samples, eliminate the need for specialized laboratory training, and improve test turnaround time. Use of feces collected from environments where wildlife reservoirs of *M*. *bovis* or other significant pathogens reside would vastly improve the ability of wildlife managers to assess the health status of wildlife, develop wildlife management strategies enhancing control of agriculturally important diseases, and provide public health support. The capability to differentiate between BCG-vaccinated and non-vaccinated animals prior to and after exposure to *M*. *bovis* would provide a crucial surveillance modality to control and eradicate bovine tuberculosis in domestic animal and wildlife reservoir populations. Continued use and refinement of our sampling method and analysis is therefore designed to lead toward development of a portable, labor and cost efficient tool that can accurately identify non-vaccinated and/or vaccinated domestic and wild animals infected with *M*. *bovis* or other pathogens of zoonotic or agricultural importance.

## Supporting information

S1 Table Study timeline from initiation of study to completion. This table documents all diagnostic and fecal sampling time-points, age at vaccination and *M*. *bovis* challenge, and all other procedures pertinent to the study beginning with purchase of calves through euthanasia and necropsy.
(TIF)

S2 Table Comparative differences in mean peak areas of sample groups. Differences in mean peak area were calculated for dependent (pre- vs. post-challenge) and independent (vaccination and infection status) comparisons. A minimum fold difference < = 3.0 criteria was used to identify suites of VOCs useful in discriminating between sample groups.
(TIF)

S1 Fig Response to PPDb by calves 104 days after virulent *M*. *bovis* challenge. Blue bars indicate skin thickness (millimeters) prior to injection of PPDb. Skin thickness 72 hours post-PPDb injection, mean, and standard deviation of measurements for non-vaccinated (NVpost) BCG-vaccinated *M*. *bovis* positive (BCGpostPOS); and BCG-vaccinated virulent *M*. *bovis* negative calves (BCGpostNEG) are indicated by red, purple and orange bars, respectively. All calves were classified as reactors based on standard interpretation of the CCT [42]. Mean responses for NVpost calves were greather than those of BCGpostPOS and BCGpostNEG calves [56].
(TIF)

S2 Fig Responses to PPDa and PPDb by calves 104 days after virulent *M*. *bovis* challenge. Blue bars represent changes in skin thickness (millimeters) 72 hours post-administration of PPDa. Differences in response to PPDb 72 hours post-administration by NVpost; BCGpostPOS; and BCGpostNEG cattle are indicated by red, purple, and orange bars, respectively. Difference in skin thickness in response to PPDa are lower than the changes noted in skin thickness in response to PPDb in all calves. Responses to PPDb are significantly greater in NVpost calves [56].
(TIF)

S3 Fig XCMS analysis sample groups using 105 statistically significant ions. Sample chromatograms are aligned and overlaid onto the x-axis. Features with high m/z ratios are represented by the dots farthest above the x-axis. Size of circles equates to degree of fold change (features with greatest fold change have the largest radii). Color intensity of the circles corresponds to the statistical significance (p-value) of the fold change as calculated by a Welch t-test with unequal variances (darker color = lower p-value).
(TIF)
