# Supplementary figures and images for: Use of fecal volatile organic compound analysis to discriminate between non-vaccinated and BCG—Vaccinated cattle prior to and after Mycobacterium bovis challenge
Source: PLoS One. 2017 Jul 7;12(7):e0179914. doi: 10.1371/journal.pone.0179914 (PMC5501492; doi:10.1371/journal.pone.0179914)

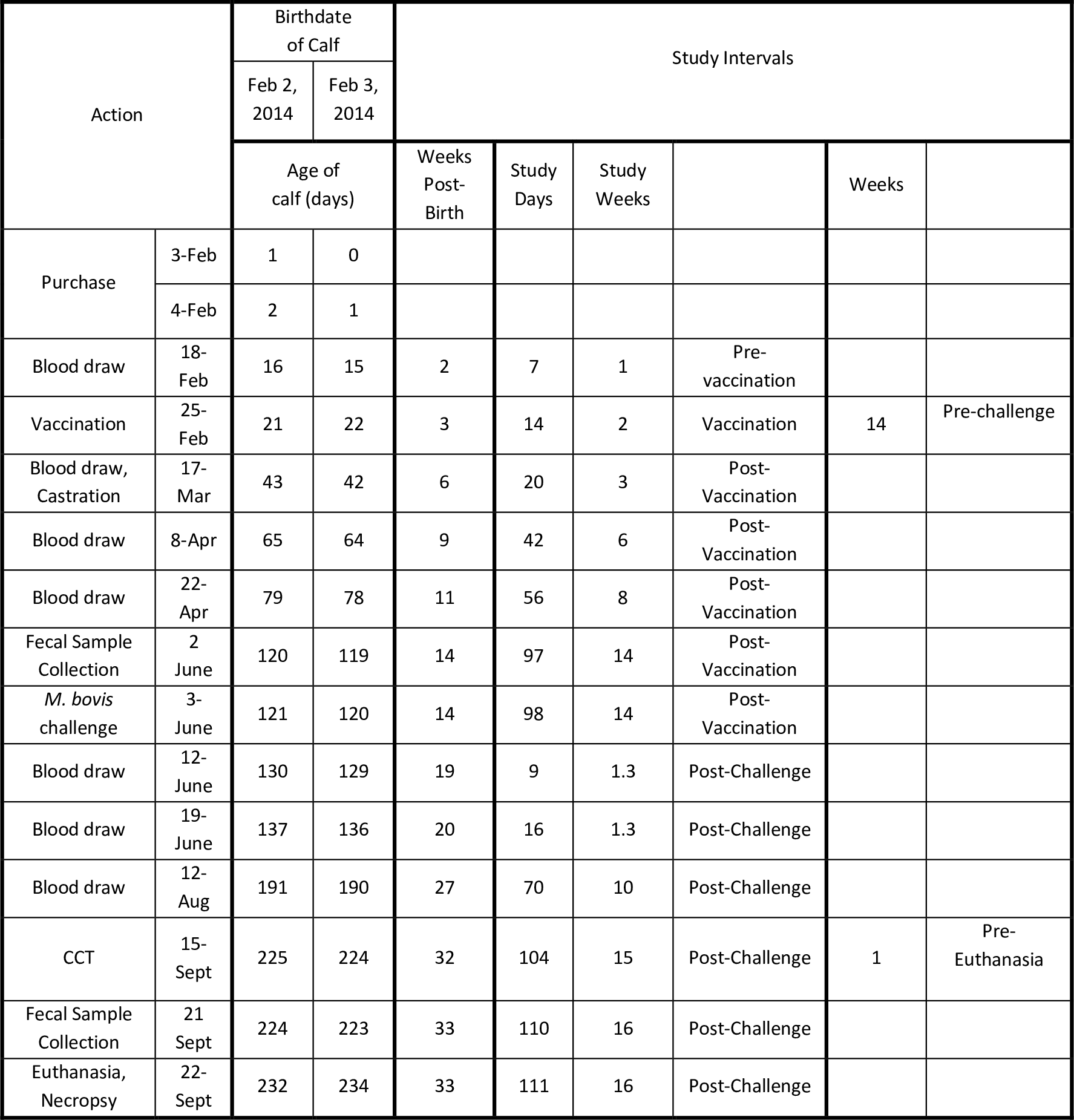

Supplement: S1 Table — This table documents all diagnostic and fecal sampling time-points, age at vaccination and M. bovis challenge, and all other procedures pertinent to the study beginning with purchase of calves through euthanasia and necropsy. (TIF) [file pone.0179914.s001.tif]

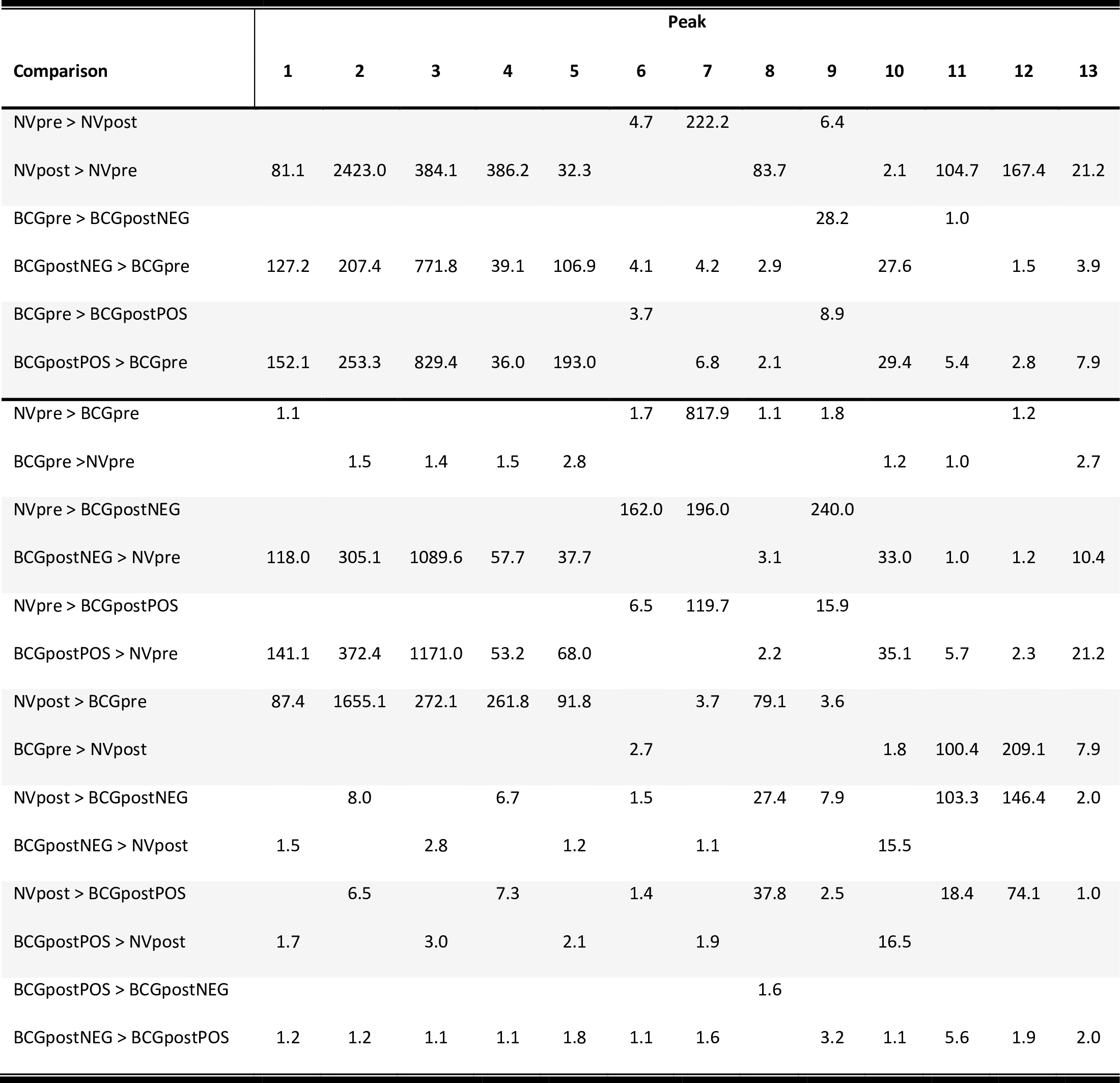

Supplement: S2 Table — Differences in mean peak area were calculated for dependent (pre- vs. post-challenge) and independent (vaccination and infection status) comparisons. A minimum fold difference < = 3.0 criteria was used to identify suites of VOCs useful in discriminating between sample groups. (TIF) [file pone.0179914.s002.tif]

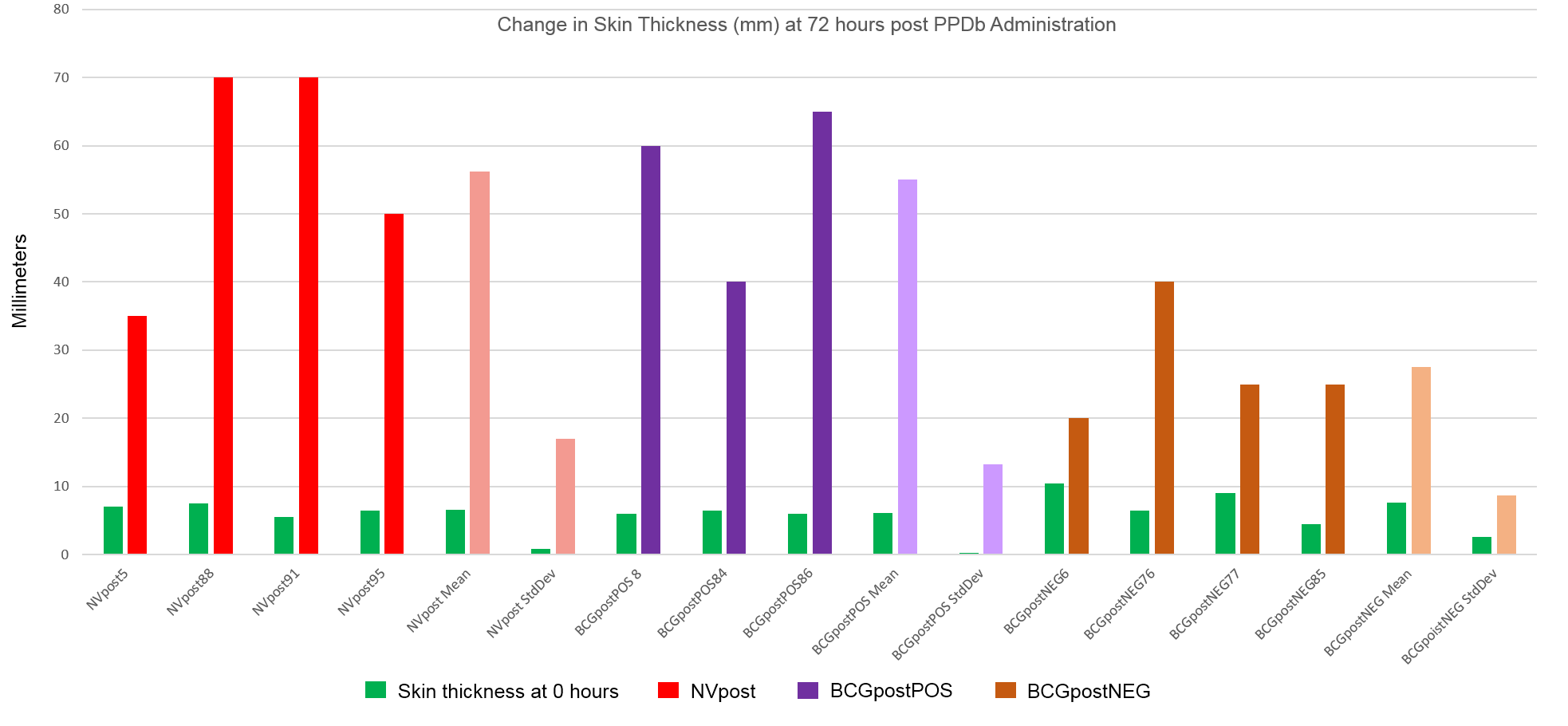

Supplement: S1 Fig — Blue bars indicate skin thickness (millimeters) prior to injection of PPDb. Skin thickness 72 hours post-PPDb injection, mean, and standard deviation of measurements for non-vaccinated (NVpost) BCG-vaccinated M. bovis positive (BCGpostPOS); and BCG-vaccinated virulent M. bovis negative calves (BCGpostNEG) are indicated by red, purple and orange bars, respectively. All calves were classified as reactors based on standard interpretation of the CCT [42]. Mean responses for NVpost calves were greather than those of BCGpostPOS and BCGpostNEG calves [56]. (TIF) [file pone.0179914.s003.tif]

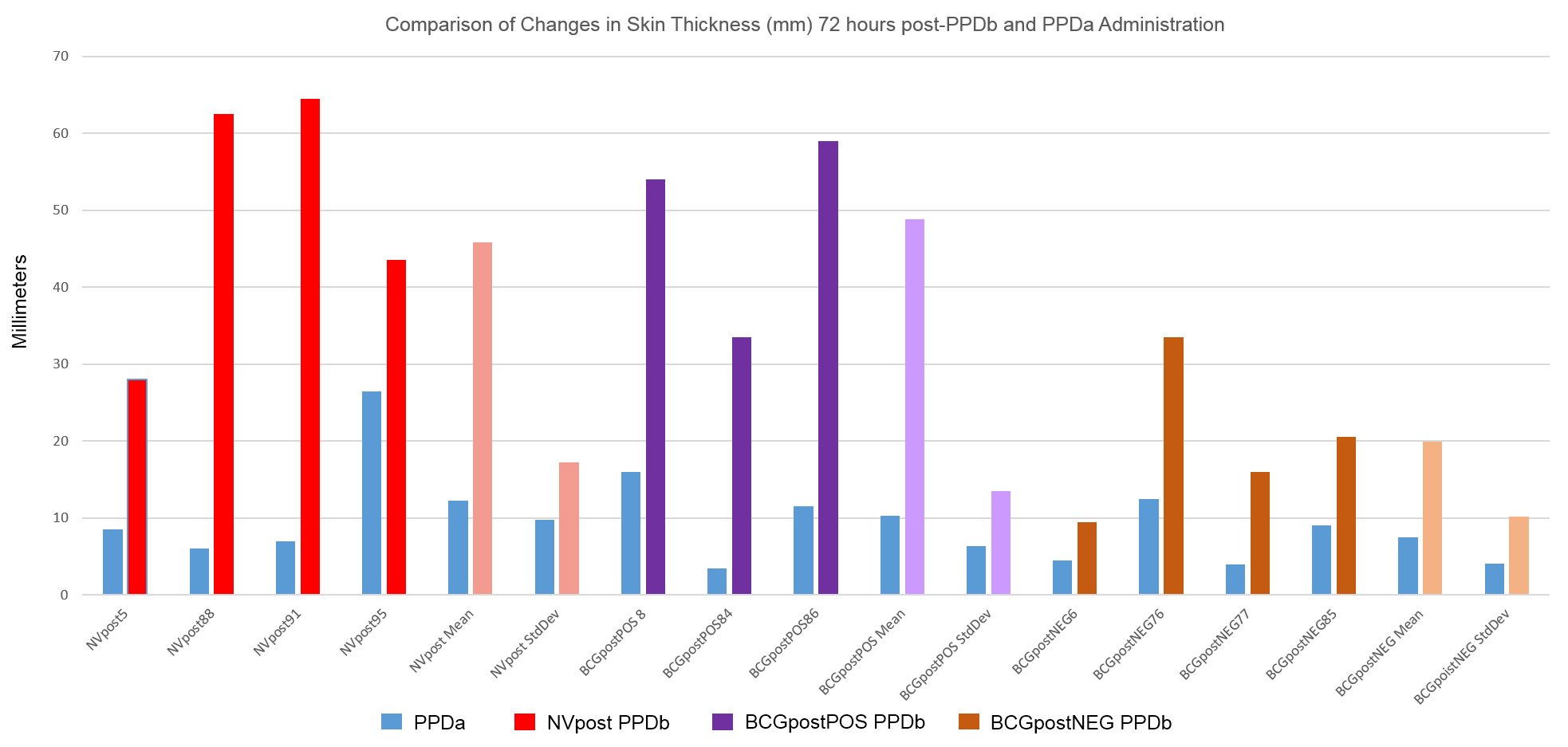

Supplement: S2 Fig — Blue bars represent changes in skin thickness (millimeters) 72 hours post-administration of PPDa. Differences in response to PPDb 72 hours post-administration by NVpost; BCGpostPOS; and BCGpostNEG cattle are indicated by red, purple, and orange bars, respectively. Difference in skin thickness in response to PPDa are lower than the changes noted in skin thickness in response to PPDb in all calves. Responses to PPDb are significantly greater in NVpost calves [56]. (TIF) [file pone.0179914.s004.tif]

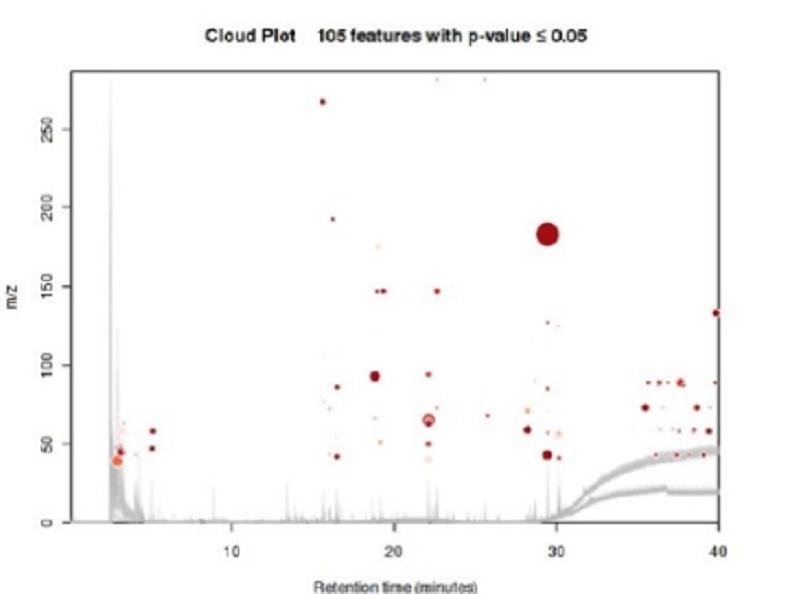

Supplement: S3 Fig — Sample chromatograms are aligned and overlaid onto the x-axis. Features with high m/z ratios are represented by the dots farthest above the x-axis. Size of circles equates to degree of fold change (features with greatest fold change have the largest radii). Color intensity of the circles corresponds to the statistical significance (p-value) of the fold change as calculated by a Welch t-test with unequal variances (darker color = lower p-value). (TIF) [file pone.0179914.s005.tif]
